# Clinical Characteristics, Management, and Outcome of the First 19 Patients With Pneumonia Due to the 2019 Novel Coronavirus Disease Treated in an Intensive Care Unit in the Republic of Cyprus

**DOI:** 10.7759/cureus.15114

**Published:** 2021-05-19

**Authors:** Antonia Kastoris, Stelios Iordanou, Christos Efseviou, Andry Papastylianou, Elpidoforos S Soteriades, Lakis Palazis

**Affiliations:** 1 Intensive Care Unit, Nicosia General Hospital, Nicosia, CYP; 2 Intensive Care Unit, Limassol General Hospital, Limassol, CYP; 3 Occupational and Environmental Medicine and Epidemiology, Harvard University, Boston, USA

**Keywords:** covid-19, mechanical ventilation, critically ill

## Abstract

Background

The widespread reach of severe acute respiratory syndrome coronavirus 2 (SARS-CoV-2) and its consequences have severely affected the consistency of healthcare systems around the world and caused millions of deaths to date. Understanding the coronavirus disease 2019 (COVID-19) manifestation, progression, and management is crucial for the healthcare personnel caring for COVID-19 patients within the intensive care unit (ICU), as well as for the patients' health progression.

Methods

A prospective observational study was used to investigate the progression of critically ill COVID-19 positive patients who were admitted to the ICU of Nicosia General Hospital from March 10 to May 1, 2020. All patients over the age of 18 were included in the study; their data were anonymously collected using the institution’s electronic medical record system and analyzed in Microsoft Excel (Microsoft Corporation, Redmond, WA). Pregnant women, children, and prisoners were excluded.

Results

During the study period, a total of 19 patients with a positive result on a reverse-transcriptase polymerase chain reaction (RT-PCR) were included in the study; 74% were men and their mean age was 64 years. Sixty-three percent of the patients were obese, 53% had a history of confirmed hypertension, 68% were admitted with severe respiratory failure, and all of them required invasive mechanical ventilation. Patients were categorized into four groups of ventilation based on the H or L ventilation phenotype in association with co-morbidities. Prone position in the first mechanical ventilation days was found to be more advantageous in L than H phenotype patients, 68% required vasopressor support, and 42% developed acute kidney injury (AKI) during their ICU stay. Diarrhea was with a median day of onset of eight days. Lactate levels above 2 mmol/L in the first four days of admission were correlated with a negative outcome. Nine patients (47%) were successfully discharged from the ICU while 10 (53%) died during their stay.

Conclusion

In critically ill patients, male gender and obesity are significant risk factors for ICU admission due to COVID-19, and early prone position, mechanical ventilation, and low positive end-expiratory pressures (PEEP) values may be beneficial, especially in the L phenotype category patients. Patients' ventilation phenotype during ICU admission and hospitalization seemed to determine the outcome.

Clinical improvement might have been higher and possibly ICU mortality lower if remdesivir was available. Hydroxychloroquine did not seem to improve patient outcomes, a consistent find, as suggested by other studies; on the contrary, it may have contributed to increased mortality rates.

## Introduction

Acute respiratory syndrome from severe acute respiratory syndrome coronavirus 2 (SARS-CoV-2), which was first detected in December 2019 in the Wuhan province of China, has already been spread in more than 100 countries worldwide. In most cases, the infection resolves with no complications. However, in other cases, it has proved to be deadly, leading to a significant need for health care systems around the world to increase their capacity of intensive care unit (ICU) beds due to the high ICU admission rates [1]. As of today, more than 126 million cases were confirmed and caused more than 2.7 million deaths worldwide (https://www.worldometers.info/coronavirus/).

The coronavirus disease 2019 (COVID-19) outbreak began in the Republic of Cyprus on March 9, 2020, with a gradual decline of positive cases until the beginning of May 2020 mainly due to the implementation of early measures of home quarantine and social distancing. Prior to the second “wave” in a population of less than 1 million, 889 positive COVID-19 cases have been identified with an overall total of 15 COVID-19 related deaths.

The Nicosia General hospital is a tertiary, referral, university-affiliated hospital with 500 operating beds, a multidisciplinary ICU with 17 beds, and a high dependency unit (HDU) with six beds in Nicosia, Cyprus. It has an average of 850 admissions per year of which 32% are scheduled surgical admissions. It has a reporting standardized mortality ratio of 0.82 and 0.88 for the ICU and HDU, respectively.

Some international reports have already been published regarding the characterization of critically ill COVID-19 patients. Further description of the progression and management of severe COVID-19 cases requiring hospitalization in ICU units would be instrumental for the decision-making processes in allocating and utilizing scarce ICU resources in different countries [2].

Aim

The aim of our study was to describe the demographic, clinical, and management characteristics, as well as the outcome, of COVID-19 patients admitted to the Nicosia General Hospital ICU unit in order to share our experience with COVID-19 critically ill patients with the wider clinical community.

## Materials and methods

Study population, setting, and data collection

Patients were identified as laboratory-confirmed COVID-19 cases that were admitted to Nicosia General Hospital, from March 10 to May 1, 2020 [3]. A confirmed case of COVID-19 was defined as a positive result on a reverse-transcriptase polymerase chain reaction (RT-PCR) assay of a specimen collected either from a nasopharyngeal swab or bronchial secretions. Only laboratory-confirmed cases were included. Patients over the age of 18 were included in the study. Pregnant women, children, and prisoners were excluded. Ethical approval for this study was provided by the Nicosia General Hospital Scientific Committee, and researchers analyzed only de-identified data from the institution’s electronic medical record system. Additional records from other public hospitals were also obtained.

Data collection: study definitions

Coexisting conditions, COVID-19 specific symptoms, and symptom duration were ascertained from physician documentation upon admission. On ICU admission, the SAPS II (Simplified Acute Physiology Score II) was also recorded.

Acute respiratory distress syndrome (ARDS) was defined as acute-onset hypoxemia (the ratio of the partial pressure of arterial oxygen to the fraction of inspired oxygen (PaO_2_:FiO_2_), <300) with bilateral pulmonary opacities on chest imaging, not fully explained by congestive heart failure or other forms of volume overload [4].

Ventilations parameters were recorded upon arrival and daily for each patient, including positive end-expiratory pressures (PEEP), PaO_2_:FiO_2_ ratio, days of mechanical ventilation, and weaning from mechanical ventilation. Patients were characterized as L type (low elastance, low lung weight, and low recruitability) or H type (high elastance, high lung weight, high recruitability) according to the definition of Dr. Gattinoni upon admission and during the length of ICU stay [5]. Data not recorded on a daily basis was not included for lack of continuous observations.

Prone positioning, tracheostomy, weaning, and critical illness neuropathy were also identified. Prone positioning was conducted in the ICU at the discretion of the attending physician. All tracheostomy procedures were conducted by ICU physicians by percutaneous technique. Successful weaning was defined as spontaneous breathing for more than three days. Critical illness neuromuscular abnormality was assessed daily by the treating physician and defined as a motor deficit or muscle weakness of bilateral and symmetric nature and most marked in the proximal muscles [6].

Vasopressor infusion was defined as the need for support of the maintenance of the set mean arterial pressure goal, after the cessation of hypnotic or opioid agents, which was sustained for more than 30 minutes, requiring an infusion of more than 0.1 μg/kg/min [7]. Noradrenaline is the vasopressor of choice. Dobutamine or levosimendan infusion was assessed at the discretion of the attending physician according to echocardiography findings and mixed venous oxygen saturation (SvO2). Acute kidney injury was defined according to the Risk, Injury, Failure, Loss of Kidney Function, and End-stage Kidney Disease (RIFLE) classification [8].

Diarrhea was defined as having three or more loose or liquid stools per day with stool volume greater than 250 ml/day [9]. *Clostridium (C.) difficile* stool samples were tested using enzyme immunoassay for the presence of toxin A/B and by high clinical suspicion with a polymerase chain reaction.

Lactate levels were recorded every 24 hours from arterial blood gas analysis and values above 2 mmol/L were recorded. Treatment included the hospital’s protocol for antiviral therapy in COVID-19 (Appendix A). ICU mortality was recorded, and all patients had a seven-day hospital follow-up.

Statistical analyses

Data were summarized using descriptive statistics presented as medians, means, and/or standard deviations as appropriate. Categorical variables were summarized as counts and percentages. Missing data were acknowledged. The analysis was performed using Excel (Microsoft Corporation, Redmond, WA).

## Results

ICU admissions due to COVID-19 included a total of 19 patients. Eleven of them were admitted to the Nicosia General Hospital (6) or other hospitals (5) due to pneumonia symptoms; three patients were admitted to other hospitals with other symptomatology. Two additional patients were admitted from home after contact with COVID-19 patients. Three patients hospitalized due to other reasons presented as in-hospital contacts.

Testing was positive from the first swab for 17 of the 19 cases, whereas in two cases, a positive test was obtained after the second or third test. For eight patients, data were available regarding the duration of COVID-19 symptoms before hospital admission with a median of seven days (range 5 to 10 days). The median time from symptom onset until mechanical ventilation was 12 days (range 6 to 19 days).

In Table 1, we present the most frequent symptoms of COVID-19 patients and their co-morbidities on hospital admission. In Table 2, we present demographic information, data on obesity, and vital signs upon ICU admission. Five patients had no known risk factors apart from obesity.

**Table 1:** Symptoms and co-morbidities of COVID-19 patients on hospital admission COPD: chronic obstructive pulmonary disease

COVID-19 symptoms	No of patients (%)
Fever	12 (63)
Cough	12 (63)
Hypoxemia	9 (47)
Malaise	5 (26)
Neurological deficits	3 (16)
Hemodynamic instability	2 (11)
Headache	2 (11)
Nausea, vomiting	2 (11)
Vertigo, dizziness	2 (11)
Loss of smell and taste	1 (5)
Rhinorrhea	1 (5)
Co-morbidities	No of patients (%)
Hypertension	10 (53)
Diabetes Mellitus type II	4 (21)
COPD	4 (21)
Current or former tobacco smoker	4 (21)
History of cardiac arrhythmias	4 (21)
Dyslipidemia	4 (21)
Chronic renal failure	3 (16)
Chronic cardiac failure	3 (16)
Hemodialysis patients	2 (11)
Previous ischaemic stroke	2 (11)
History of coronary artery disease	2 (11)
Pacemaker	2 (11)
History of cancer	1 (5)
Recent surgery	1 (5)
Other	7 (37)
None	5 (26)

**Table 2:** Demographic information and vital signs of patients upon ICU admission (N=19) BMI: body mass index, SAPS II: simplified acute physiology score, bpm: beats per minute, PaO_2_:FiO_2_: ratio of the partial pressure of arterial oxygen to fraction of inspired oxygen

Demographic Information	
Age: Mean (range)	64 (29-78)
Males	14 (74)
BMI: Mean (range) kgm-^2^	28 (19 – 37)
BMI (kgm^-2^) n (%)	
BMI > 35	2 (11)
BMI 30 to 35	6 (31.5)
BMI 25 and 30	4 (21)
BMI 20 to 25	6 (31.5)
BMI < 20	1 (5)
SAPS II score: Mean (range)	53 (20 – 92)
Intubation before admission n (%)	12 (63)
Vital Signs upon ICU admission	
Vital sign	n (%)
Temperature > 38^o^C	2 (11)
Heart rate > 100 bpm	6 (31.5)
Vasopressor infusion	8 (42)
Respiratory rate > 20	9 (47)
PaO_2_:FiO_2_ < 150	13 (68)
PaO_2_:FiO_2_150 – 250	4 (21)
PaO_2_:FiO_2_ > 250	2 (11)

Upon admission, no patient had coinfection with influenza or respiratory syncytial virus. One of the patients had coinfection with an enterovirus - rhinovirus. All bacterial cultures were negative for bacterial growth upon admission.

Ventilation and respiratory parameters

All patients received invasive mechanical ventilation. Only one patient received noninvasive ventilation (NIV) prior to intubation in the ICU. Three patients were admitted to the ICU prior to having received NIV.

Extubation was performed successfully in six patients with a median of 5.5 days (range 4 to 9 days). Weaning in four tracheostomy patients was successful between the 18^th^ and 23^rd^ days of ICU stay. Ventilation and respiratory parameters are illustrated in Table 3.

**Table 3:** Description of ICU course and outcome of 19 COVID-19 patients in Cyprus NA: not applicable, T: tracheostomy, MV: mechanical ventilation, E: extubation, TP: T piece, PP: prone position, PNT: pneumothorax, PEEP: positive end-expiratory pressure, ECMO: extracorporeal membrane oxygenation, NOEC: no escalation of care, VAP: ventilator-associated pneumonia. PaO_2_:FiO_2_: ratio of the partial pressure of arterial oxygen to fraction of inspired oxygen

Patient Number	Ventilation phenotype admission	Ventilation phenotype during the stay	Median compliance (range) (L cmH_2_O^-1^)	Days of MV	Median PEEP (range) (cmH_2_O)	Lowest PaO_2_:FiO_2_ [day of MV]	Highest PaO_2_:FiO_2_ [day of MV]	Outcome
1	L TYPE	L TYPE	No continuous data	30	7 (5 – 12)	129 [day 4] – 93 [day 14 - sepsis]	309 [day 12]	BRAIN DEATH - Death
2	L TYPE	L TYPE	No continuous data	22	6 (5 – 10)	120 [day 2]	370 [day 16]	Death -82 days
3	L TYPE	L TYPE	No continuous data	17	6 (5 – 7)	230 [day 1]	334 [day 9]	DEATH
4	L TYPE	L TYPE	No continuous data	12	6 (5 – 8)	67 [day 2]	155 [day 12]	DEATH - NOEC
5	L TYPE	L TYPE	No continuous data	12	6 (5 – 8)	283 [day 1]	354 [day 7]	Death – 32 days
6	H TYPE	L TYPE	No continuous data	21	7 (5 -13)	85 [day 3]	260 [day 19]	T P – Discharge
7	H TYPE	L TYPE	38 (30 – 52)	28	8.5 (6 – 14)	96 [day 4] -64 [day 21 -VAP]	218 [day 18]	Death – 69 days
8	L TYPE	L TYPE	No continuous data	21	6 (5 – 14)	109 [day 4]	400 [day 13]	TP - Discharge
9	L TYPE	L TYPE	No continuous data	4	5.5 (5 – 6)	196 [day 1]	265 [day 4]	E – Discharge
10	L TYPE	L TYPE	54 (52 -57)	8	8 (6 – 12)	97 [day 2]	216 [day 3] PP	E – Discharge
11	L TYPE	L TYPE	45 (42 -55)	3	6 (6 – 8)	100 [day 1]	264 [day 4]	E – Discharge
12	H TYPE	H TYPE	23 (19 – 28)	14	9 (6 – 12)	55 [day 14 - sepsis]	200 [day 8] PP	DEATH (15 DAYS ICU)
13	L TYPE	L TYPE	No continuous data	17	6 (5 – 8)	130 [day 1]	343 [day 15]	TP - Discharge
14	L TYPE	L TYPE	40	2	2 (PNT)	191 [day 3]	288 [day 1]	DEATH (2 DAYS ICU)
15	L TYPE	L TYPE	No continuous data	2	9 (8-10)	93 [day 2]	153 [day 1]	DEATH (1 DAY ICU)
16	H TYPE	H TYPE	33 (29 – 39)	10	12.5 (12 – 16)	63 [day 1]	84 [day 8] PP	DEATH (10 DAYS ICU)
17	L TYPE	L TYPE	50 (42 -55)	5	10 (6-12)	75 [day 1]	221 [day 2] PP	E – Discharge
18	H TYPE	L TYPE	No continuous data	9	8 (6-10)	57 [day 5]	191 [day 6] PP	E- Discharge
19	H TYPE	H TYPE	26 (22 -33)	6	12 (10-13)	40 [day 6]	216 [Day 2] PP	ECMO Day 6 – Discharge

Tracheostomy, weaning, and critical illness neuropathy

A total of nine (47%) of the 19 patients underwent percutaneous tracheostomy. One of the nine patients underwent urgent tracheostomy due to intubation failure. Six patients received a late tracheostomy. All these patients exhibited critical illness neuropathy. The two early tracheostomy patients did not exhibit signs of neuropathy.

Prone position

A total of six patients (31.5%) were positioned in the prone position. Four of the six patients exhibited H-type phenotypes (three during the whole course of their stay, whereas one changed to the L phenotype). The patient with a change in phenotype had a positive outcome. Two of the three true H types (2 died with severe respiratory failure, whereas the last patient was placed on extracorporeal membrane oxygenation (ECMO)). Two L phenotype patients improved greatly with the prone position strategy after two sessions of prone positioning.

Hemodynamic status

Eight patients (42%) required vasopressor support upon admission without signs of a new infection. Thirteen patients (68%) required vasopressor support during their stay, with five patients having signs of a new infection. Troponin levels upon admission were negative in all patients and no positive troponin level was recorded during the length of stay. Only one patient received both dobutamine and levosimendan infusion.

Renal function

Two patients had chronic kidney failure. Eight patients (42%) exhibited acute kidney injury (AKI) during their stay according to the RIFLE criteria; six requiring continuous renal replacement therapy (CRRT). Of these eight patients with AKI during the stay, three (38%) exhibited AKI upon admission to the ICU. Of the remaining five patients that did not exhibit signs of AKI on admission, three progressed into kidney failure requiring CRRT, whereas two exhibited kidney injury without the need for CRRT.

Gastrointestinal symptoms

Seven patients (37%) had diarrhea during their stay. The day of onset ranged between the third and 26^th^ days with a median of eight days. The duration of symptoms was between three and seven days. Stool samples for C. difficile were sent in all patients (in two patients twice (one PCR testing) and were negative. One patient had a previous medical history of irritable bowel syndrome. No abdominal pain, nausea, or vomiting, in addition to diarrhea, could be documented due to the depressed level of consciousness associated with their disease severity.

Lactate levels

A total of 10 (53%) of the 19 patients had lactate levels above 2 mmol/L upon admission to the ICU. Lactate levels on admission did not correlate with the outcome. Nevertheless, it appeared that patients with lactate levels above 2 mmol/L in the first four days of admission (and during the whole stay) had a worse outcome. Due to the small number of ICU admissions, this observation is not statistically significant.

Antiviral treatment

A treatment protocol was adhered to for all COVID-19 critically ill patients (Appendix A). Remdesivir was not available at the time of the study in our hospital. All patients received corticosteroids. In total, 10 (53%) of the 19 patients received tocilizumab. No positive or negative correlation was found between the administration of tocilizumab and disease outcome. Patients receiving tocilizumab were more critically ill.

Outcomes

A total of nine (47%) of the 19 patients were discharged from the ICU and the hospital, and 10 patients (53%) died in the ICU. On one patient, ECMO was used. Patient outcomes with days of mechanical ventilation and/or tracheostomy/extubation are summarized in Table 3.

From the 10 fatalities, eight died due to severe respiratory distress and two due to sepsis. One patient had serious aortic valve stenosis, pneumothorax, and shock. One patient died shortly after extubation (without signs of respiratory distress) from shock and cardiac arrest. One patient became brain dead on the 22^nd^ day. The average age of the deceased patients was 69 years, ranging from 58 to 78 years. These patients died between the second and 30^th^ day of ICU admission (median of 13 days). Two deaths occurred within two days of ICU admission.

## Discussion

To our knowledge, this is the first study in the Republic of Cyprus examining the course of disease progression among COVID-19 severe cases admitted in the ICU.

Despite a relatively small number of patients, our study adds important observations regarding the suggested risk factors for COVID-19 such as obesity. In addition, we have identified that lactate levels in the first four days of admission above 2 mmol/L correlated with a negative outcome and a clinical management approach regarding mechanical ventilation.

We reported a median of 12 days from illness onset until invasive mechanical ventilation, similar to reports in the literature. A relatively slow deterioration primarily of lung function and a relatively protracted course has been previously reported [10]. Common symptoms included cough and fever [11]. Three patients presented with neurological deficits. Previous studies show that neurological symptoms that manifest as an acute cerebrovascular disease with impaired consciousness and skeletal muscle injury may be present [12].

Risk factors were predominantly male gender, age, and arterial hypertension as described in the international literature [13-14]. The male gender tends to have a higher risk of severe infection and mortality than the female, which may be associated with the increased prevalence of cardiopulmonary disease and smoking, as well as the protective effect of elevated estrogen levels in females. Nevertheless, the differences between male and female mortality are still under investigation [15].

We observed that many of the admitted patients in our ICU were obese. There may be a correlation between the severity of illness and weight especially in younger males with no smoking or previous medical history. A recent study in France concluded that disease severity increased with body mass index (BMI) and identified obesity as a risk factor for COVID-19 infection [16].

We identified four categories of ARDS patients as per Dr. Gattinoni’s classification of ventilation phenotypes [5,17]: (1) L phenotype patients seemed to benefit from a few days of mechanical ventilation. These patients suffered from minor or no comorbidities; (2) L phenotype patients seemed to suffer from ICU complications and had a prolonged weaning process. These patients had several comorbidities and/or older age; (3) H phenotype patients that eventually changed to L phenotype. Among this group, the patients exhibited radiological and ultrasonographic signs of atelectasis [18]. Prone positioning seemed of great benefit to these patients. This group exhibited obesity with a high waist circumference partially explicatory of the high atelectasis rate found; (4) “Classic” ARDS patients or pure H phenotype. They had the worst outcome since they suffered from severe ARDS.

Furthermore, we observed that the L type during mechanical ventilation seemed to benefit most from lower PEEP values than the ARDS network [19] suggests and could endure larger tidal volumes during ventilation of even up to 8 ml/kg without changes in plateau pressures. We reported lower median PEEP values used than in previous studies in Italy [2]. The H type, on the other hand, seemed more difficult to ventilate, in the notion of a “classic ARDS,” requiring higher PEEP values and smaller tidal volumes to maintain satisfactory plateau pressures. Prone positioning also did not seem to change the outcome in these patients since oxygenation improved temporarily whilst in the prone position.

A proportion of patients were admitted due to hemodynamic instability. It seems that some patients do not show severe ARDS symptoms but shock as the main reason for ICU admission [20]. Several mechanisms may play a role, namely, direct damage by the virus, destabilization of coronary plaques, microthrombogenesis, systemic inflammatory responses, and aggravated hypoxia [21]. Interestingly three patients requiring vasopressor infusion exhibited normal echocardiography findings. A possible explanation is that COVID-19 utilizes the angiotensin-converting enzyme 2 (ACE2) receptor for viral cell entry [22]. ACE2 catalyzes the conversion of angiotensin II to angiotensin 1-7, which acts as a vasodilator [22]. This may lead to hemodynamic instability without direct myocardial involvement. In this context, a patient (no previous history of cardiovascular disease) who was extubated without signs of respiratory distress went into shock and cardiac arrest. During the arrest, echocardiography revealed a grossly decreased left ventricular function without dilatation of the right ventricle. No autopsy was performed. A possible explanation could be myocardial infarction as also previously mentioned by Zhou et al. [23].

Acute renal injury following COVID-19 infection has been reported with a high prevalence in the international literature. The renal impairment caused by the infection is most likely multifactorial and diverse and may be partially explained due to the presence of the virus in the kidney entering through ACE 2 receptors, inflammation caused by the disease, or through secondary effects seen in critically ill patients. Kidney injury is associated in these patients with a higher risk of in-hospital death [24].

Gastrointestinal symptoms were also reported in our patients with a high prevalence. Diarrhea was the main gastrointestinal symptom observed during ICU stay, which may be related to the COVID-19 activity, the antibiotics or/and antiviral drugs used, as well as to the cytokine storm generated by COVID-19 [25-26]. Nevertheless, the exact cause of diarrheal remains unclear.

Treatment with tocilizumab, a blocker of Interleukin-6, in patients with a cytokine storm may affect inflammation and cellular viral entry [27-28]. Nevertheless, half of the patients who had received tocilizumab experienced severe sepsis up to 20 days after administration, whereas the other half of the patients improved immensely.

Treatment with remdesivir, a nucleotide analog prodrug that inhibits viral ribonucleic acid (RNA) polymerases, was not administered since it was not available in our hospital at the time of the study. Published literature suggests that our patient's mortality (52.6%) might have been lower using remdesivir. A study published later than our study period resulted in a 13% mortality, on a similar age (median 67y) of severe COVID-19 patients receiving invasive mechanical ventilation [29]. The comparison between the mortality percentages is noteworthy; therefore, remdesivir could be used to update the current institutional treatment protocol.

The antimalarial drug hydroxychloroquine (HCQ) gathered attention that it can control the cytokine storm and shorten the clinical recovery time among critically ill patients. In the current study, 400 mg of HCQ (daily) was administered to all patients following the Food and Drug Administration (FDA) emergency use authorization for COVID-19 at that time. Later clinical evidence showed a lack of benefit, as well as an increased risk of QT interval prolongation and potentially lethal ventricular arrhythmias [30]. Therefore, HCQ was excluded from the current institutional treatment protocol for COVID-19.

Limitations

Our study has several notable limitations. It is a small study within a relatively short period of follow-up. Some clinical data, which may have clinical importance, were incomplete, missing, or not documented. Finally, since the sample size provided is small, it can limit the generalizability, and no conclusion can be drawn on the effectiveness of the therapy provided due to the lack of statistical power.

## Conclusions

In conclusion, COVID-19 patients exhibit a large heterogeneity of symptoms and course of disease during ICU stay. Neurological manifestations of the illness should not be overlooked. Obesity seems to be a considerable risk factor for unfavorable outcomes. The ventilation phenotype on admission and during ICU stay appears to have predictive power over the course of the disease and may be classified into four types of ventilation. Also, lactate levels recorded during the first four days of admission may play a role in disease outcome. Mechanical ventilation, lower PEEP values, and early prone positioning may provide more favorable outcomes, especially in L phenotype patients. Bigger studies could provide further insight into the course of disease and outcome in critically ill patients.

## Appendices

**Table 4:** Treatment protocol ARDS: acute respiratory distress syndrome, PaO_2_:FiO_2_: ratio of the partial pressure of arterial oxygen to fraction of inspired oxygen, WBC: white blood cells

Medication	Dosage and Duration	Indication
Azithromycin	500 mg for 7 Days	All patients
Hydroxychloroquine	400 mg twice daily for the first day followed by 200 mg twice daily for 10-14 days	All patients
Lopinavir/Ritonavir	400/100 mg twice daily for 10-14 days	All patients
Tocilizumab	Dose 8 mg/kg (maximum dose 800 mg/dose) Once or if no satisfactory response twice	Definition of critical illness and/or “cytokine storm” indication ARDS Pao2:Fio2 < 300 mmHg, Ferritin > 1000 ng/ml (or increasing values), D-dimer > 1000 ng/ml, Polymorphonuclear leukocyte (or total WBC)/Lymphocytes > 3-3,5, Thrombocytopenia, IL-6 > 40 pg/ml
Antimicrobial therapy	Ceftriaxone 2000 mg daily or empirically according to physician discretion	All patients
Low molecular weight heparin	LMW heparin 40 mg/day. If the patient is overweight or obese, increase dose at 1 mg/kg/day	All patients
Corticosteroids	Methylprednisolone 1 mg/kg daily for 5 days then dose tapering or dexamethasone 20 mg daily then tapering	Recommended
